# Dislocation of Ankle, with Compound Fracture of the Tibia and Fibula

**Published:** 1863-07

**Authors:** James S. King

**Affiliations:** Lemont, Illinois; Formerly Resident Physician St. John’s Hospital, Cincinnati, Ohio


ARTICLE XVIII.

### DISLOCATION OF ANKLE, WITH COMPOUND FRACTURE OF THE TIBIA AND FIBULA.

Reported by JAMES S. KING, M.D., of Lemont, Ill. Formerly Resident-Physician St. John's Hospital, Cincinnati, Ohio.

July 24th, 1862.—Was called with Dr. Hall, of this place, to see M. S., a healthy Irish boy, æt. 20, reported to have broken his leg. On arriving at his residence, we found the limb in the following condition:—The tibia protruding about two inches through a transverse lacerated wound, extending about an inch on either side of internal malleolus, which remained *in situ* upon astragalus being fractured from tibia. Fibula fractured about two inches above articulation, lower fragment being driven inwards and upwards; upper portion of bone protruding through a longitudinal lacerated wound of about an inch and a-half in length, commencing just above external malleolus and extending upwards; foot turned outwards and at nearly a right angle with leg; anterior and posterior tibial arteries uninjured. Patient's general condition good,—pulse but little accelerated; and he seemed in good spirits notwithstanding the severity of his injuries, caused by the falling of a horse upon him in the quarry, about one hour previous to our seeing him. His ankle was caught between the side of the horse and the sharp corner of a rubble stone.

As his parents were decidedly opposed to having his limb amputated, we determined to try conservative surgery upon the case; and proceeded to reduce dislocation, having first placed the patient fully under the influence of chloroform. We had considerable trouble to get the parts into proper position, but at last succeeded, without sawing any of the bones. Placed limb in Pennsylvania Hospital fracture-box; ordered cold water dressing; left opiates to allay pain; and requested consultation for next day, which being consented to, we telegraphed for Dr. Dagget, of Lockport.

July 25th.—Found patient feverish,—pulse about 100; complains of great pain in ankle, notwithstanding the opiates. Gave him morphine, nitre, and ipecac; ordered a cathartic. Dr. DAGGET thought we had best continue cold water dressing to limb,—thought that the limb could be saved.

July 26th.—Patient about as yesterday. Continued treatment. His mother now raised a hue and cry about the doctor's bill. Said, she would not have two doctors visiting him every day. The case was passed to my care.

July 27th.—Patient less feverish; wound looks well; does not complain of so much pain.

July 31st.—I have visited patient every day since 27th, he is doing well,—but very little febrile action; but little pain in ankle,—wound in good condition. The old lady now declared I should not visit the boy any more until she sent for me. I tried to convince her of the necessity of my attendance, but she persisted in her determination, and I left the case to nature and an Irish woman,—very incompatible agents by the way.

August 5th.—Was sent for in great haste: boy reported very bad. I refused to go, except in company with Dr. HALL, to which they very reluctantly consented,—the doctor's bill being the bugbear. We found patient with high febrile action,—pulse 120; limb hot and swollen; wound covered with unhealthy looking pus; patient restless and complaining of great pain in joint. The old lady had discontinued the use of water dressing after we left the case. We dressed the limb; ordered warm applications to wound; gave patient Doveri, ipecac, and nitre; ordered lemon-water and beef-essence. Requested that Dr. DAGGET should see the case on next day.

August 6th.—Repaired to house of patient, in company with Dr. DAGGET. Prepared to amputate limb, if condition of patient was not better than on yesterday. On arriving, found patient comparatively comfortable; but little febrile action; limb not so much swollen,—suppurating freely; appearance, every way an improvement upon yesterday. On consultation, we determined to try conservative surgery a while longer. We gave the old lady full directions as to what she must do, after which, she concluded that she could get along without us,—said, she would send for me if she needed me. I told her, I would visit the boy as I thought best or not at all, to which she would not consent, and I left the case.

December 11th.—M. S. came into my office, walking without crutches. Upon examination of limb, found the foot somewhat turned inwards upon ankle, caused by the limb having been allowed to lie in that position in fracture-box. Says, he has but little pain in joint, but that it it very weak. Found an opening of about a-half of an inch in extent over internal malleolus, from which there is a constant but small discharge of pus, caused by the presence of dead bone which he refused to let me remove; also found a small opening over region of fracture upon the outer side of limb, from which he says he took quite a large piece of bone about a month ago. He has been walking without crutches about six weeks. After I left the case, he had no treatment, except that his mother had followed the directions we had left; and he had been to Lockport once since he was able to walk.

July 3d, 1863.—M. S. came into my office. On examination of limb, found foot in about same position it was last December. Motion at ankle-joint antero-posteriorally about one-fourth of what it is in normal condition of joint, laterally, about as of other limb. Wound over internal malleolus entirely healed; says, that several pieces of bone came out of the wound about a week after I saw him in December. Upon examination of the bones which he gave me, I found them to be the internal malleolus, together with splinters of bone from tibia. Wound upon exterior of limb still open, from which there is a small discharge of pus, caused by some portions of dead bone, but, as it does not pain him, will not permit me to remove it. He now gave me the bone which came out of his wound last November; find it about half of an inch in length, and to be a portion of the lower fragment of fibula. He says, he now works on the farm, finds joint still weak, but it is improving all the time; sometimes has considerable pain in it, evidently, of a rheumatic character. Says, he would not give the present limb for a hundred wooden ones.

